# Low miR-214-5p Expression Correlates With Aggressive Subtypes of Pediatric ALCL With Non-Common Histology

**DOI:** 10.3389/fonc.2021.663221

**Published:** 2021-05-25

**Authors:** Piero Di Battista, Federica Lovisa, Enrico Gaffo, Ilaria Gallingani, Carlotta C. Damanti, Anna Garbin, Lavinia Ferrone, Elisa Carraro, Marta Pillon, Luca Lo Nigro, Rossella Mura, Marco Pizzi, Vincenza Guzzardo, Angelo Paolo Dei Tos, Alessandra Biffi, Stefania Bortoluzzi, Lara Mussolin

**Affiliations:** ^1^ Division of Pediatric Hematology, Oncology and Stem Cell Transplant, Maternal and Child Health Department, University of Padova, Padova, Italy; ^2^ Istituto di Ricerca Pediatrica Città della Speranza, Padova, Italy; ^3^ Department of Molecular Medicine, University of Padova, Padova, Italy; ^4^ Center of Pediatric Hematology Oncology, Azienda Policlinico G. Rodolico – San Marco, Catania, Italy; ^5^ Department of Paediatric Haematology-Oncology, Ospedale Pediatrico Microcitemico, Cagliari, Italy; ^6^ Surgical Pathology and Cytopathology Unit, Department of Medicine - DIMED, University of Padova, Padova, Italy; ^7^ CRIBI Interdepartmental Research Center for Innovative Biotechnologies (CRIBI), University of Padova, Padova, Italy

**Keywords:** ALCL, childhood, miRNA, prognosis, biomarker, RNA-seq

## Abstract

The unsatisfactory cure rate of relapsing ALK-positive Anaplastic Large-Cell Lymphoma (ALCL) of childhood calls for the identification of new prognostic markers. Here, the small RNA landscape of pediatric ALK-positive ALCL was defined by RNA sequencing. Overall, 121 miRNAs were significantly dysregulated in ALCL compared to non-neoplastic lymph nodes. The most up-regulated miRNA was miR-21-5p, whereas miR-19a-3p and miR-214-5p were reduced in ALCL. Characterization of miRNA expression in cases that relapsed after first line therapy disclosed a significant association between miR-214-5p down-regulation and aggressive non-common histology. Our results suggest that miR-214-5p level may help to refine the prognostic stratification of pediatric ALK-positive ALCL.

## Introduction

Anaplastic Large-Cell Lymphoma (ALCL) accounts for 10-15% of pediatric and adolescent non-Hodgkin lymphomas. Most pediatric ALCL cases carry ALK gene fusions (ALK-positive ALCL) that constitutively activate RAS-ERK, JAK3-STAT3 and PI3K–Akt oncogenic signaling pathways, thus promoting cancer cell proliferation, differentiation, and survival (1). In pediatric ALK-positive ALCL, the current treatment regimens achieve a progression-free survival of ~70% at 10 years from diagnosis (2). The prognosis of patients with resistant or relapsing disease is still relatively poor (3), calling for an increased understanding of ALCL aggressiveness and for the development of new prognostic markers for the early identification of patients with higher risk of treatment failure, who could benefit from more intensive chemotherapy regimens. Minimal Disseminated Disease (MDD), measured by PCR detection of NPM-ALK, and low anti-ALK antibody titer at diagnosis are significantly associated with inferior outcome (2). Recently, a large multicentric study confirmed that non-common (NC) histological patterns (e.g. small cell or lympho-histiocytic ALK-positive ALCL) are key relapse risk factors, either considered alone or in combination with MDD (2). Previous studies suggested an important role of microRNAs (miRNAs) in adult onset ALCL, and compared the ALK-positive and ALK-negative phenotypes, identifying miR-17~92 cluster up-regulation in ALK-positive ALCL and miR-155 overexpression in ALK-negative cases (4). For the first time, this study associates the risk of relapse in pediatric ALCL with miRNA signatures and identifies miRNA expression patterns specifically related to high risk NC histotypes.

## The Small RNA Landscape of Pediatric ALCL Shows Dysregulation of Several miRNAs

Small RNA (sRNA) sequencing (**Supplementary Methods**) was performed on a set of 20 pediatric ALK-positive ALCL cases. Six reactive lymph node (RLN) biopsies were also analyzed as controls. An extended cohort of 58 ALCL cases and 10 RLN, including 39 independent ALCL cases and 4 RLN, was used to validate the differential expression of selected miRNAs (**Supplementary Table 1**). All patients were treated according to the ALCL-99 protocol (2). The study was approved by the local institutional ethical committee and informed consent was obtained from the patients’ legal guardians. The sRNA-seq data were analyzed with miR&moRe2 v0.2.3 (5) and DESeq2 to test for differential expression between ALCL and RLN samples (**Supplementary Methods**). Of the 1,013 miRNA-derived sRNAs detected, 449 miRNAs and 24 miRNA-offset RNAs (moRNAs) expressed in all samples of at least one group were compared. Only 51 miRNAs (5% of the detected) accounted for 95% of the observed sRNA expression (**Supplementary Figure 1B**). MiR-21-5p, miR-148a-3p and let-7i-5p were amongst the top expressed miRNAs in ALCL.

**Figure 1:**
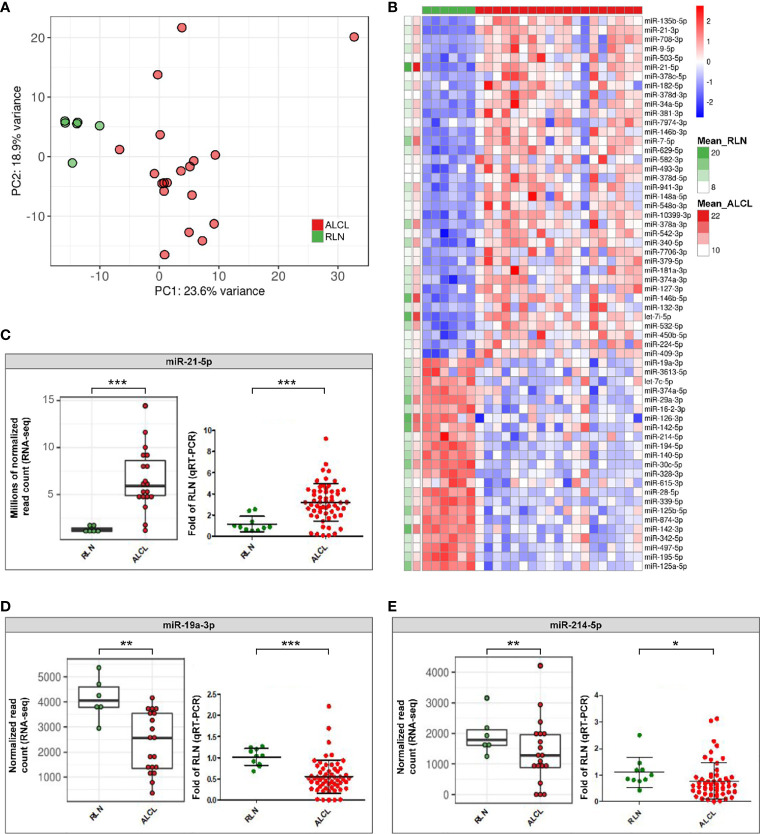
Differentially expressed sRNAs in primary tumours of ALCL patients compared to reactive lymph nodes (RLN). **(A)** Unsupervised principal component analysis of sRNA expression profiles in ALCL (red dots) and RLN (green dots) samples. **(B)** Heatmap of the 60 miRNAs most dysregulated in ALCL compared with RLN (q-value ≤ 0.01 and absolute log2 Fold Change (LFC) ≥ 1; standardized expression; the two columns on the left indicate the mean expression in ALCL and RLN samples, for each miRNA); **(C–E)** Expression of miR-21-5p, miR-19a-3p and miR-214-5p determined by RNA-seq in the discovery cohort (left panel; ** and *** q-value < 0.01 and < 0.001) and validated by qRT-PCR in the extended cohort (right panel; * and ***, p-value < 0.05 and < 0.001; Mann-Whitney).

Interestingly, ALCL and RLN had different sRNA expression profiles (**Figure 1**) and 121 miRNAs resulted significantly deregulated (q-value ≤ 0.01) in ALCL (**Supplementary Table 2**). **Figure 1** shows the 60 most dysregulated (q-value ≤ 0.01 and absolute fold change ≥ 2) miRNAs, of which 37 were up-regulated and 23 were down-regulated in ALCL. Dysregulation of some of these miRNAs was previously described in adult ALCL primary tumors or cell lines (4), whereas most have not been reported before. In line with our results, miR-135b-5p is up-regulated in ALK-positive ALCL tumor biopsies compared to RLN (6) and miR-142-5p, miR-142-3p and miR-29a-3p are down-regulated compared to peripheral T cells of healthy donors (7). Quantification by qRT-PCR in the extended cohort confirmed the significant up-regulation of miR-21-5p (Mann Whitney, p-value = 0.0005; **Figure 1**), and the decreased expression of miR-19a-3p (p-value = 0.0001; **Figure 1**) and miR-214-5p (p-value = 0.02; **Figure 1**) in ALCL compared to RLN.

MiR-21-5p is a known oncomiR up-regulated in aggressive activated B cell-type diffuse large B-cell lymphomas compared to germinal center B cell-type cases (8). Previous data indicated that miR-21-5p is more expressed in ALCL than in the less mature T-cell acute lymphoblastic leukemia cell lines (9), raising the hypothesis that the up-regulation of miR-21-5p could reflect an activated T-cell phenotype of tumor cells. We examined also activated T-cells from healthy donors (**Supplementary Figure 2**), an alternative normal counterpart and putative cell-of-origin of ALCL. Quantification in activated T-cells confirmed the significant upregulation of miR-21-5p in ALCL (Mann Whitney, p-value = 0.02; **Supplementary Figure 3A**). From a pathobiological perspective, this evidence further corroborated our finding about the ectopic expression of this oncomiR in ALCL, whose role in the malignant transformation warrants further investigation.

Next, miR-19a-3p significantly downregulated in ALCL compared to RLN (**Figure 1**) was demonstrated to be significantly reduced also in comparison to activated T-cells (Mann Whitney, p-value = 0.047; **Supplementary Figure 3B**), suggesting that miR-19a-3p could play a tumor suppressor role in ALCL, in line with recent data in invasive breast cancer (10).

## MiR-214-5p Is Down-Regulated in ALK-Positive ALCL With NC Histology and High Risk of Relapse

**Figure 2:**
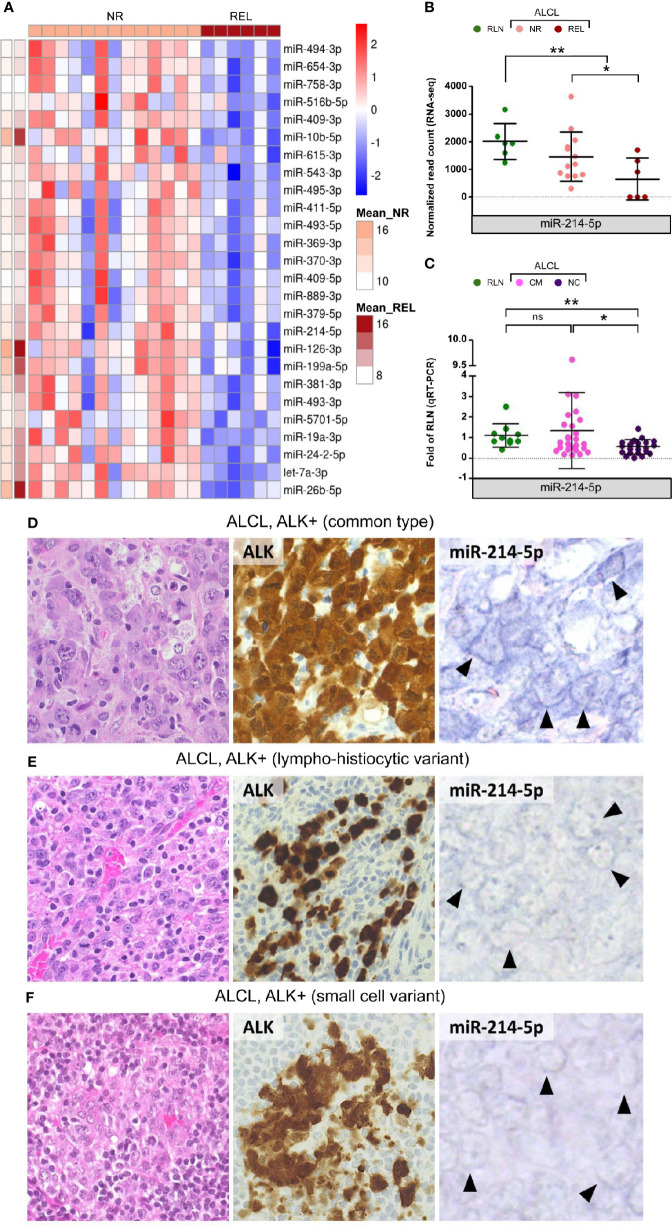
Differentially expressed miRNAs among relapsed and non-relapsed ALCL cases. MiR-214-5p expression is significantly reduced in ALCL of non-common histological subtypes compared to common histology. **(A)** The 26 significantly down-regulated miRNAs in relapsed (REL) compared to non-relapsed (NR) ALCL cases of the discovery cohort (Mean_REL and Mean_NR average expression (normalized read count) in REL and NR patients, respectively); **(B)** MiR-214-5p expression is significantly lower in REL compared with NR samples and in ALCL patients of the discovery cohort with respect to reactive lymph nodes (RLN), according to sRNA-seq (* and ** q-value < 0.05 and < 0.01, respectively). **(C)** MiR-214-5p expression quantification by qRT-PCR in the extended cohort of ALCL patients with common (CM) and non-common (NC) histology and in RLN from healthy donors (data relative to RLN; * and ** p-value < 0.05 and 0.01, respectively; Mann Whitney, ns, not significant). **(D)** CM ALK + ALCL (n = 6), histologically characterized by sheets of large, atypical cells with sternbergoid features, include neoplastic elements strongly and diffusely positive for ALK1 (cytoplasmic and nuclear expression), with moderate to strong positivity for miR-214-5p by *in situ* hybridization (5/6 tested cases; positive signal: blue staining in tumor cell cytoplasm - *arrowheads*). ALK+ ALCL lympho-histiocytic **(E)** and small cell **(F)** NC variants (n = 6) are instead characterized by weak to absent cytoplasmic signal for miR-214-5p (faint blue signal – *arrowheads*) (H&E and peroxidase stain; original magnification, x 40).

To explore the usefulness of miRNAs in disease risk stratification, miRNA expression was assessed at diagnosis in patients that underwent relapse after first line therapy. Compared to non-relapsed (NR) cases, relapsing (REL) ALK-positive ALCL samples were characterized by the down-regulation of 26 miRNAs (**Figure 2**). These included miR-10b, miR-126-3p, miR-199a-5p, and miR-26b-5p within the most expressed in ALCL. Of note, miR-214-5p (**Figure 2**), miR-615-3p, miR-126-3p and miR-19a-3p (**Supplementary Figure 4**), which are down-regulated in ALCL, resulted significantly less expressed also in REL compared to NR patients. Since the NC histology is associated with a worse prognosis (2), we examined miR-214-5p expression in patients of the extended cohort. Compared to common type ALCL (CM), cases with NC histology disclosed significantly lower expression of miR-214-5p (Mann Whitney, p-value = 0.046; **Figure 2**). This observation was confirmed by *in situ* hybridization for miR-214-5p on representative tissue sections from ALCL cases with CM (n = 6) and NC (n = 6; 4 lympho-histiocytic and 2 small cell) histology. High positivity for miR-214-5p was observed for 5/6 CM ALCL and in none of the 6 NC variants (p-value = 0.02; **Figure 2D** and **Supplementary Table 3**). The association between miR-214-5p down-regulation and NC histology is of particular interest since NC histological patterns are currently not considered among the factors for risk-based treatment stratification. Estimating miR-214-5p expression level by qRT-PCR and miRNA *in situ* hybridization (11) could prove useful to complement the standard histopathological evaluation of pediatric ALK-positive ALCL at diagnosis.

## Discussion

Low miR-214-5p expression is associated with poor prognosis in other solid tumors (12, 13), whereas high miR-214-5p expression with good outcome in diffuse large B-cell lymphoma (14). Beyond its prognostic value, the biological implications of miR-214-5p deregulation in ALK-positive ALCL with NC histology are poorly characterized and deserve further investigation. MiR-214-5p down-regulation can play a key role in ALCL lymphomagenesis, since it may lead to the de-repression of several oncogenic targets, thus driving increased cell proliferation and tumor cell migration (12, 13, 15). This is in line with previous studies in other malignancies, whereby the impairment of miR-214-5p by competitor endogen RNAs (e.g. DANCR, LINC00963 and circ-XPR1) promotes cancer cell proliferation and metastasis (12, 13, 15). These observations may be even more valid for ALK-positive ALCL, since miR-214-5p physiologically blocks key pathways for ALCL proliferation, migration and invasion. Indeed, miR-214-5p dampens Notch pathway activation by repressing the JAG1 Notch ligand (16). Notch signaling is of key importance for ALK-positive ALCL pathobiology, being constitutively activated by the NPM-ALK fusion gene through STAT3 (17). In summary, this study provides an unprecedented view of the miRNAome in pediatric ALK-positive ALCL highlighting the up-regulation of miR-21-5p, and the down-regulation of miR-19a-3p and miR-214-5p. Our results also highlight miRNAs that are particularly down-regulated in high-risk cases. Of importance, the uncovered link between miR-214-5p down-regulation and ALCL with NC histology and unfavorable prognosis suggests the use of selected miRNAs as novel biomarkers to complement the standard prognostic stratification of pediatric ALK-positive ALCL at diagnosis.

## Data Availability Statement

The datasets presented in this study can be found in online repositories. The names of the repository/repositories and accession number(s) can be found below: Gene Expression Omnibus, accession number GSE171323.

## Ethics Statement

The studies involving human participants were reviewed and approved by the ethics committee for clinical experimentation of the Padova hospital CESC (Comitato Etico per la Sperimentazione Clinica Azienda Ospedaliera di Padova). Written informed consent to participate in this study was provided by the participants’ legal guardian/next of kin.

## Author Contributions

PB, FL, SB and LM conceived the study. FL, IG, AG and CD selected and processed clinical samples and obtained sequencing data. AG and LF processed buffy coats from healthy donors and performed T-cell activation. PB and SB contributed bioinformatics methods and data analysis. PB and IG performed miRNAs quantification by qRT-PCR. EC and MPil collected clinical data and revised the manuscript. MPiz and VG collected histological samples, performed in situ hybridization analysis. LN, RM, AD and AB provided patients clinical care. PB, FL, AG, SB, EG and LM wrote the manuscript. PB, FL, AG and MPiz made the figures. LM supervised the experimental work. All authors contributed to the article and approved the submitted version.

## Funding

This work has been supported by Associazione Italiana per la Ricerca sul Cancro, Milano, Italy (Investigator Grant – IG 2018 #21385 to LM and IG 2017 #20052 to SB); Fondazione Città della Speranza (grant 19/03 to LM); Fondazione CARIPARO, Padova, Italy (grant 17/03 to LM); Italian Ministry of Education, Universities and Research (PRIN 2017 #2017PPS2X4_003 to SB); Fondazione Roche per la Ricerca, Rome, Italy (grant to FL) and Fondazione Umberto Veronesi, Milano, Italy (fellowship to EG).

## Conflict of Interest

The authors declare that the research was conducted in the absence of any commercial or financial relationships that could be construed as a potential conflict of interest.
