# Supplementary material for: Low miR-214-5p Expression Correlates With Aggressive Subtypes of Pediatric ALCL With Non-Common Histology
Source: Front Oncol. 2021 May 25;11:663221. doi: 10.3389/fonc.2021.663221 (PMC8185221; doi:10.3389/fonc.2021.663221)
Supplement: Supplementary file 1 [file DataSheet_1.pdf]

Low miR-214-5p expression correlates with aggressive subtypes of pediatric ALCL with non-common histology

Piero Di Battista[1,2,§], Federica Lovisa[1,2,§], Enrico Gaffo[3], Ilaria Gallingani[1,2], Carlotta C. Damanti[1,2], Anna Garbin[1,2], Lavinia Ferrone[1,2], Elisa Carraro[1], Marta Pillon[1], Luca Lo Nigro[4], Rossella Mura[5], Marco Pizzi[6], Vincenza Guzzardo[6], Angelo Paolo Dei Tos[6], Alessandra Biffi[1,2], Stefania Bortoluzzi[3,7,*], Lara Mussolin[1,2,*]

[1] Division of Pediatric Hematology, Oncology and Stem Cell Transplant, Maternal and Child Health Department, University of Padova, Padova, Italy.

[2] Istituto di Ricerca Pediatrica Città della Speranza, Padova, Italy.

[3] Department of Molecular Medicine, University of Padova, Padova, Italy.

[4] Center of Pediatric Hematology Oncology, Azienda Policlinico G. Rodolico – San Marco, Catania, Italy.

[5] Department of Paediatric Haematology-Oncology, Ospedale Pediatrico Microcitemico, Cagliari, Italy.

[6] Surgical Pathology and Cytopathology Unit, Department of Medicine - DIMED, University of Padova, Padova, Italy.

[7] CRIBI Interdepartmental Research Center for Innovative Biotechnologies (CRIBI), University of Padova, Padova, Italy.

§ these authors contributed equally

* co-last authors

Correspondence:

Lara Mussolin, Ph.D

Assistant Professor

Division of Pediatric Hematology, Oncology and Stem Cell Transplant

Maternal and Child Health Department

Padova University

Via Giustiniani 3

35128 Padova, Italy

e-mail lara.mussolin@unipd.it

Keywords: ALCL, childhood, miRNA, prognosis, biomarker

Supplementary Material

Supplementary Methods

Small RNA sequencing

Total RNA was isolated from biopsies using Trizol reagent (ThermoFisher Scientific), following the manufacturer's instructions, and quantified using a Nanodrop 1000 spectrophotometer (ThermoFisher Scientific). The RNA quality was assessed on an Agilent 2100 Bioanalyzer (Agilent Technologies) and only samples with RIN ≥ 5 were further processed for sRNA-seq. Small RNA cDNA libraries were prepared from 2 µg of total RNA using the NEBNext Multiplex Small RNA Library Prep Kit for Illumina (New England Biolabs), following the manufacturer's instructions. Sequencing was performed on an Illumina HiSeq 4000 platform to produce single-end reads (50 nt) with an average depth of 45 M reads.

Small RNA detection and quantification

RNA-seq data were analyzed by miR&moRe2 v0.2.3 (1,2), processing each sample as follows: after adapter trimming by Cutadapt v2.5 (3), good quality reads (mean base Qphred > 30, maximum two bases with Qphred < 20 and length range between 15 and 31 nt) were mapped with Bowtie v1.1.2 (4) to the human genome (GRCh38) and to know pre-miRNA sequences, including 30 nucleotides flanking pre-miRNA genes in the genome to also consider miRNA-offset RNAs (moRNAs). DESeq2 v1.24.0 (5) was used to normalize read count data and to test for differential expression, considering significant Benjamini-Hochberg adjusted p-value ≤ 0.05. The sva package (6) was applied to remove batch effects in each comparison.

T-lymphocytes isolation and activation

T-lymphocyte have been isolated by density gradient centrifugation using Lymphoprep™ (StemCell). Buffy coats from three healthy donors were layered on Lymphoprep™ in a 1:2 ratio and centrifuged at 820 x g for 15 minutes without brake. In order to obtain T-lymphocytes avoiding their activation, peripheral blood mononuclear cells from buffy coats were labeled with CD45 and CD19 antibodies. Cells were then sorted and CD45+/CD19- cells were activated using PMA (20 ng/µl, Sigma-Aldrich) and ionomycin (1 µg/ml, Sigma-Aldrich) for 4 hours. RNA was extracted using Norgen total RNA isolation kit (Norgen Biotek Corp.) and T-cells activation was confirmed by qRT-PCR of IFN-γ, IL-4 and IL-10 expression relative to untreated cells by means of comparative delta Ct method ($2^{-\Delta\Delta Ct}$). Actin-β was used as housekeeping gene (Supplementary Figure 2).

MiRNA expression quantification by qRT-PCR

Quantification of miRNAs by real-time PCR (qRT-PCR) was performed using the TaqMan Advanced miRNA cDNA Synthesis Kit and the specific TaqMan Advanced miRNA assays for miR-21-5p (ID 477975_mir), miR-19a-3p (ID 479228_mir) and miR-214-5p (ID 478768_mir) (ThermoFisher Scientific), in accordance with the manufacturer's protocol. The relative miRNA expression was quantified by means of comparative delta Ct method (2-ΔΔCt) in respect to RLN or activated T-lymphocytes, using the geometric mean of the three less variable miRNAs (let-7f-5p, miR-16-5p and miR-191-5p) as calibrators. GraphPad (GraphPad Prism, version 7.0 for Windows) software was used for statistical analysis of qRT-PCR data. Mann-Whitney test (p-value ≤ 0.05) was used to compare miRNA relative expression between different conditions.

MiR-214-5p *in situ* hybridization

Tissue sections from 6 common type (CM) ALK+ ALCL cases and 6 ALK+ ALCL non-common (NC) histological variants (4 lymphohistiocytic and 2 small cell ALK+ ALCL) were digested with *in situ* hybridization (ISH) protease 1 (Ventana Medical Systems, Milan, Italy) and treated with 95% ethanol before air-drying. The slides were pre-hybridized for 1 hour with SH buffer (Ambion) before overnight incubation at 49–56 °C in buffer containing the 5′-biotin-labeled miR-214-5p miRCURY™ LNA detection probe (Exiqon, Woburn, MA, USA). After washing in Tris-buffered saline with Tween (TBST) and GenPoint™ stringent wash solution (54 °C for 30 min), the slides were exposed to H2O2 blocking solution (DakoCytomation) and to a further blocking buffer (DakoCytomation). They were subsequently stained with primary streptavidin–horseradish peroxidase (HRP) antibody, biotinyl tyramide, secondary streptavidin–HRP antibody, and DAB chromogen solutions. Hematoxylin was used to slightly counterstain each case. MiR-214-5p expression was assessed only considering the blue cytoplasmic signal of neoplastic cells and semi-quantitatively scored as follows: score 0 = no signal; score 1 = weak expression; score 2 = moderate expression; score 3 = strong expression. miR-214-5p expression levels were attributed by comparison with internal endothelial cell controls (7). Comparison between CM and NC ALCL cases was performed by Fisher's exact test, collapsing the attributed scores into two major categories: (i) low miR-214 expression (score 0 and score 1) cases; and (ii) high miR-214 expression (*i.e.* score 2 and score 3) cases. Differences were considered statistically significant for p-value < 0.05 (**Supplementary Table 3**).

Supplementary Figures and Tables

Supplementary table 1. Clinical characteristics at diagnosis of the ALCL study cohorts.

Characteristic	RNA-seq set (N=20)	Validation set (N=58)
**Gender**		
Female	7 (35%)	21 (36%)
Male	13 (65%)	37 (64%)
**Age (years)**		
< 10	10 (50%)	28 (48%)
≥ 10	10 (50%)	30 (52%)
**Stage at diagnosis**		
I-II	2 (10%)	10 (17%)
III-IV	18 (90%)	48 (83%)
**Relapse**		
Yes	6 (30%)	21 (36%)
No	14 (70%)	37 (64%)
**Median follow-up time (years)**	4.1	3.7
**Median time to relapse (years)**	0.6	0.6
**Histology**		
Common	10 (50%)	28 (48.3%)
Small cell	-	1 (1.7%)
Mixed with small cell	2 (10%)	9 (15.5%)
Lymphohistiocytic	2 (10%)	8 (13.8%)
Mixed with lymphohistiocytic	-	2 (3.4%)
Hodgkin-like	-	2 (3.4%)
Unknown	6 (30%)	8 (13.8%)

Supplementary Figure 1. MiRNA expression according to RNA-seq. (A) Cumulative expression of 1,013 miRNA-derived small RNAs detected in ALCL (red line) and **(B)** RLN samples (green line), ordered by expression level. MiRNAs corresponding to the cumulative expression percentage are reported in Supplementary Table 1.

A

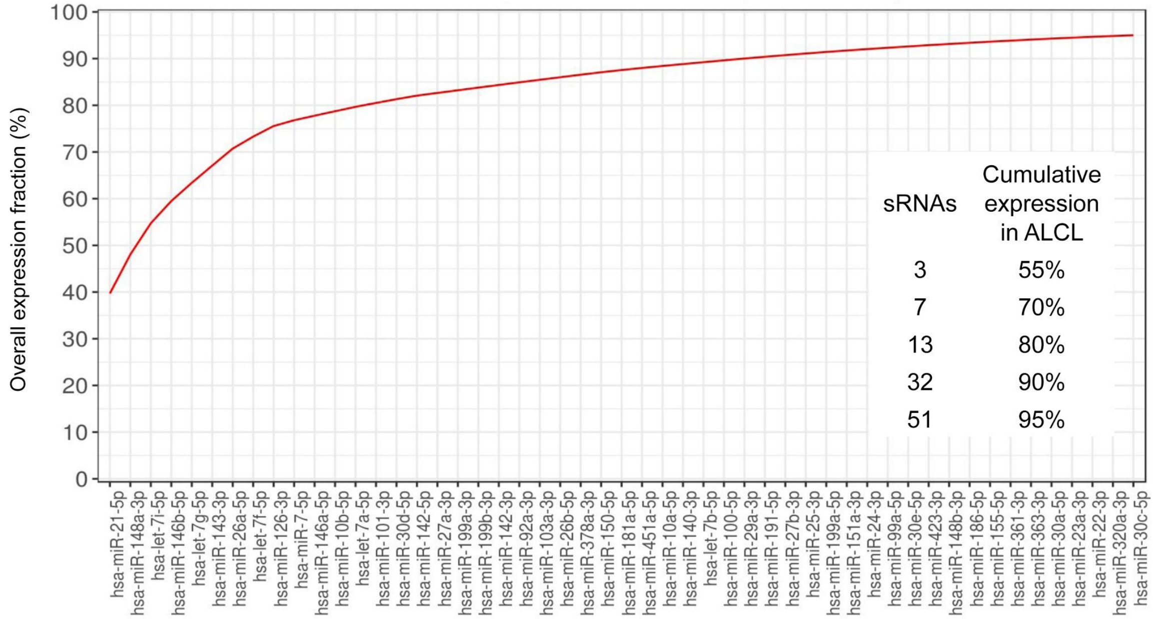

B

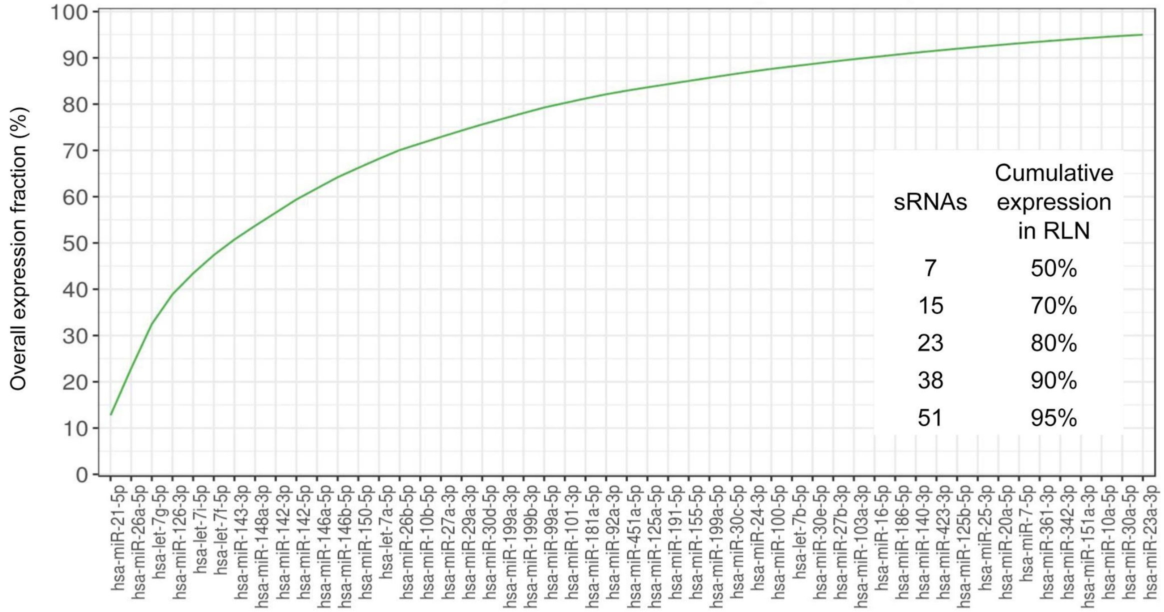

Supplementary Figure 2. T-lymphocytes activation by PMA/ionomycin treatment of peripheral CD45+/CD19- cells from healthy donors. Expression levels of (A) IFN-γ, (B) IL-4 and (C) IL-10 in T-lymphocytes after 4 hours of PMA/ionomycin treatment compared to untreated cells.

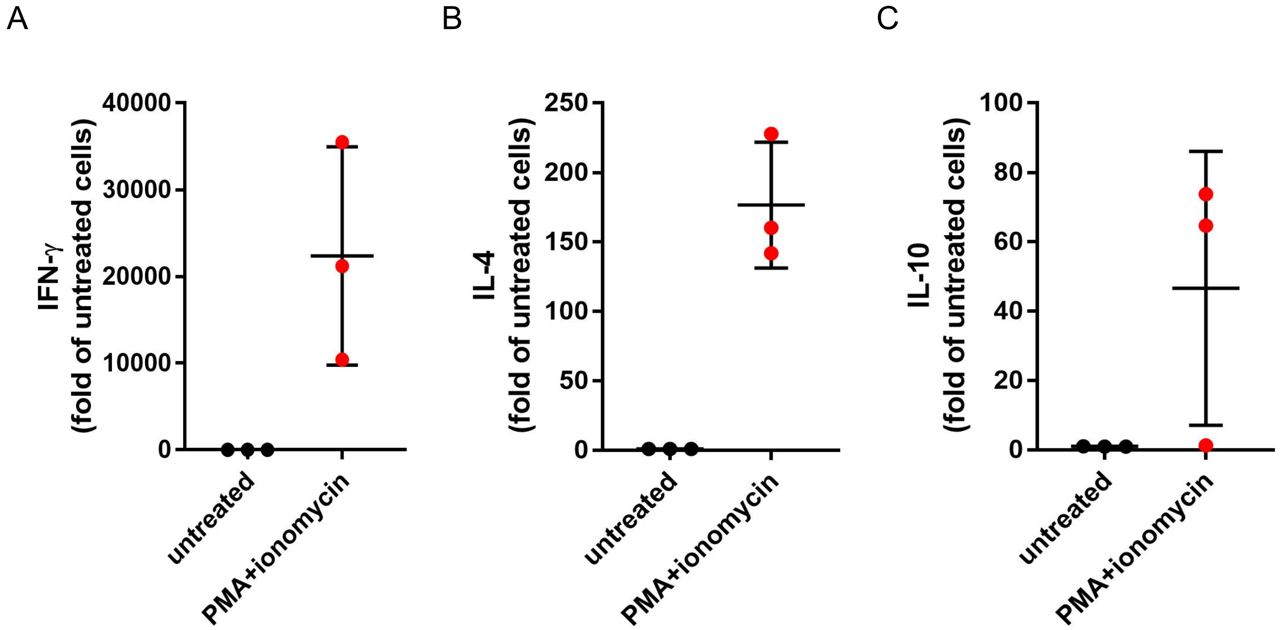

Supplementary Figure 3. MiR-21-5p and miR-19a-3p quantification by qRT-PCR in the extended cohort of ALCL patients compared to activated T-cells (data relative to activated T-cells; Mann Whitney, *p-value < 0.05).

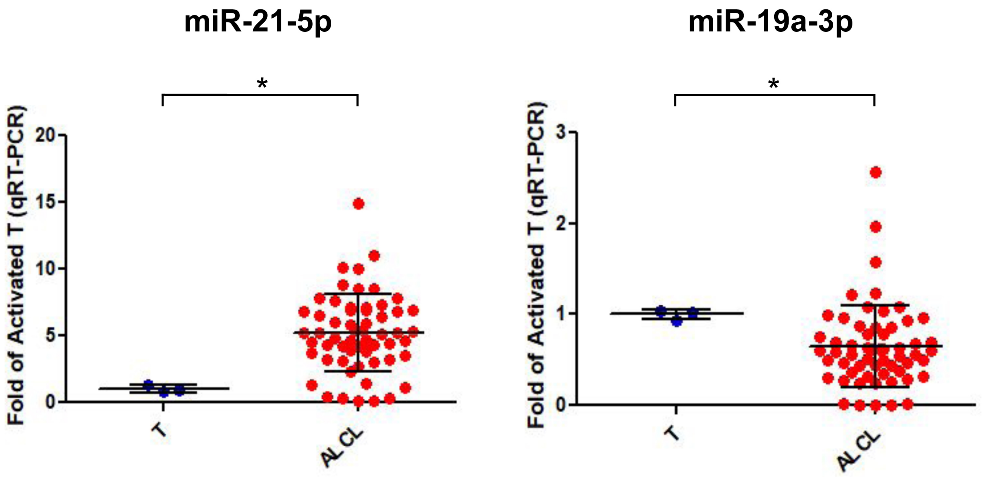

Supplementary Figure 4. MiRNAs with reduced expression in ALCL compared to RLN and in REL compared with NR ALCL. Expression levels of 3 miRNAs significantly lower in REL compared with NR samples and in ALCL patients with respect to RLN, according to sRNA-seq (*q-value < 0.05, **q-value < 0.01).

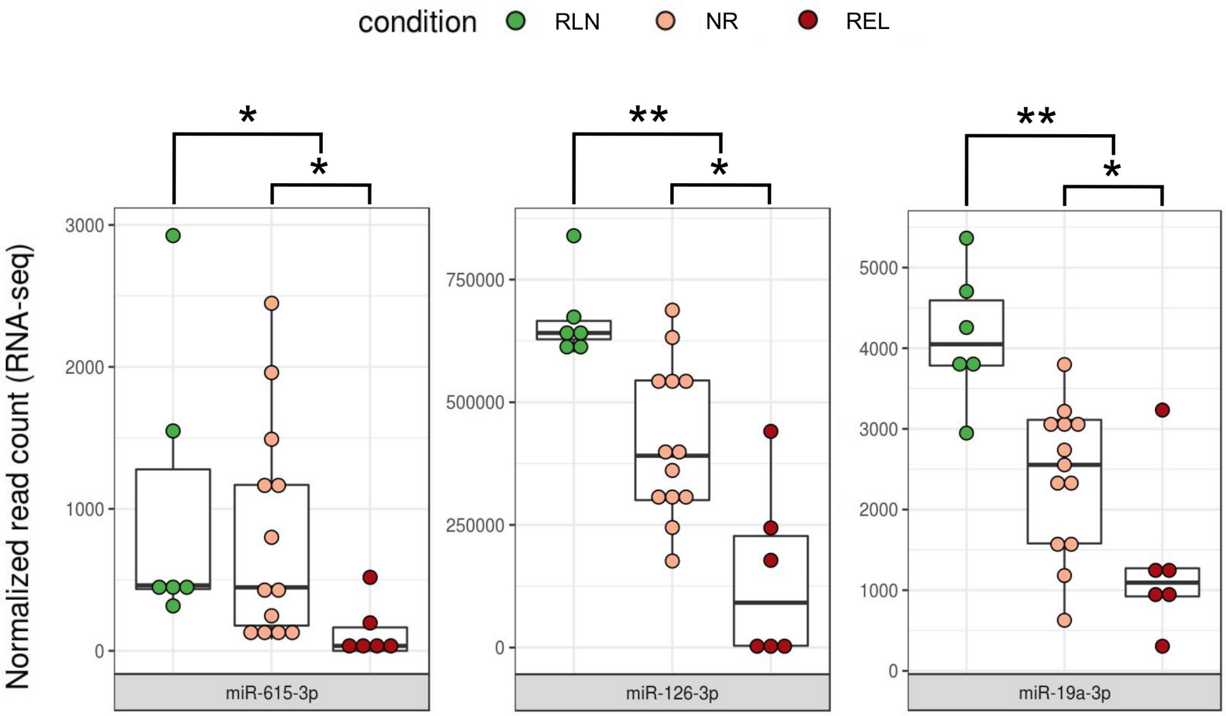

Supplementary Table 2. The 121 dysregulated miRNAs in ALCL biopsies compared to RLN. For each miRNA the expression level in ALCL patients and RLN as the average normalized read count, the fold change in log2 scale (LFC) between the groups and the p-value corrected for multiple tests (q-value) are reported.

miRNA	ALCL expression	RLN expression	LFC	q-value
miR-4425-3p	467	2	4.7	8.4×10^{-11}
miR-135b-5p	3,279	158	3.6	1.99×10^{-11}
miR-3614-5p	137	5	3.5	6.52×10^{-8}
miR-21-3p	16,799	861	3.5	8.44×10^{-21}
miR-147b-3p	462	24	3.0	1.07×10^{-6}
miR-708-3p	3,210	275	2.8	7.53×10^{-5}
miR-9-5p	13,051	1,754	2.6	2.2×10^{-6}
miR-503-5p	925	158	2.1	8.75×10^{-6}
miR-21-5p	6,617,917	1,329,320	2.1	8.4×10^{-11}
miR-378c-5p	2,153	120	2.0	9.74×10^{-5}
miR-182-5p	7,499	1,202	2.0	8.06×10^{-4}
miR-378d-3p	1,563	73	2.0	1.45×10^{-5}
miR-34a-5p	6,516	1,436	2.0	4.01×10^{-6}
miR-381-3p	5,781	1,030	2.0	1.87×10^{-6}
miR-7974-3p	1,368	151	2.0	3.65×10^{-3}
miR-146b-3p	7,168	1,688	1.9	2.69×10^{-7}
miR-889-3p	250	44	1.9	1.85×10^{-5}
miR-7-5p	194,827	39,523	1.9	6.78×10^{-7}
miR-629-5p	11,848	1,037	1.9	1.06×10^{-11}
miR-582-3p	1,633	250	1.8	9.9×10^{-4}
miR-493-3p	688	145	1.8	3.89×10^{-4}
miR-378d-5p	892	75	1.8	5.42×10^{-5}
miR-3158-3p	362	68	1.8	1.09×10^{-4}
miR-941-3p	13,162	3,413	1.8	4.72×10^{-6}
miR-152-5p	97	15	1.7	5.06×10^{-3}
miR-148a-5p	3,028	716	1.7	2.91×10^{-4}
miR-212-5p	232	40	1.7	8.06×10^{-4}
miR-548o-3p	911	267	1.7	1.58×10^{-4}
miR-127-5p	217	45	1.6	1.29×10^{-4}
miR-10399-3p	899	286	1.6	4.25×10^{-8}
miR-584-5p	101	33	1.6	5.26×10^{-3}
miR-378a-3p	77,107	16,924	1.5	7.63×10^{-5}
miR-542-3p	816	250	1.4	9.32×10^{-5}
miR-340-5p	21,498	7,451	1.4	7×10^{-9}
miR-7706-3p	684	228	1.4	1.68×10^{-3}

miR-500a-5p	205	74	1.4	3.39×10^{-5}
miR-379-5p	1,372	360	1.4	2.24×10^{-3}
miR-181a-3p	5,818	2,321	1.3	7.68×10^{-4}
miR-10399-5p	174	68	1.3	6.69×10^{-4}
miR-374a-3p	4,279	2,016	1.3	1.07×10^{-5}
miR-127-3p	8,579	2,868	1.2	2.12×10^{-3}
miR-146b-5p	748,991	250,484	1.2	3.97×10^{-6}
miR-134-5p	362	88	1.2	9.81×10^{-3}
miR-132-3p	2,637	1,026	1.1	4.73×10^{-3}
let-7i-5p	1,055,880	473,665	1.1	2.15×10^{-8}
miR-532-5p	20,339	9,283	1.1	5.92×10^{-8}
miR-450b-5p	765	364	1.1	1.46×10^{-3}
miR-224-5p	1,766	660	1.1	2.64×10^{-3}
miR-409-3p	1,434	345	1.1	3.87×10^{-3}
miR-185-3p	472	204	1.0	4.03×10^{-3}
miR-92b-3p	2,605	1,278	1.0	3.48×10^{-8}
miR-4677-3p	122	62	1.0	5.13×10^{-4}
miR-148b-3p	40,857	23,050	1.0	2.18×10^{-5}
miR-99b-3p	1,368	547	0.9	1.62×10^{-4}
miR-152-3p	18,765	8,205	0.9	2.4×10^{-3}
miR-1307-5p	830	227	0.9	9.01×10^{-3}
let-7f-1-3p	430	275	0.9	3.38×10^{-4}
miR-589-5p	896	365	0.8	5.41×10^{-3}
miR-652-3p	4,456	1,386	0.8	4.8×10^{-4}
miR-106b-3p	10,083	5,475	0.8	1.33×10^{-4}
miR-320a-3p	23,350	12,158	0.8	4.73×10^{-3}
miR-103a-3p	83,513	51,523	0.7	5.26×10^{-3}
miR-423-5p	19,726	12,443	0.6	2.34×10^{-3}
miR-28-3p	19,738	12,103	0.6	1.31×10^{-3}
let-7a-5p	145,953	206,628	-0.5	5.47×10^{-3}
let-7a-3p	2,462	3,214	-0.5	1.34×10^{-4}
let-7g-5p	619,166	999,973	-0.6	5.41×10^{-3}
miR-1271-5p	244	390	-0.6	5.46×10^{-3}
miR-191-5p	54,382	72,549	-0.6	1.53×10^{-4}
let-7d-5p	11,584	15,355	-0.7	7.47×10^{-3}
miR-423-3p	37,288	44,991	-0.7	2.39×10^{-4}
miR-26a-5p	590,314	1,059,210	-0.7	3.74×10^{-3}
miR-24-3p	49,178	67,695	-0.7	1.53×10^{-5}
miR-128-3p	14,648	19,218	-0.7	8.75×10^{-6}
miR-484-5p	5,492	8,614	-0.8	9.74×10^{-5}
miR-15b-5p	1,966	3,822	-0.8	8.24×10^{-3}

miR-766-3p	353	329	-0.8	4.54×10^{-3}
miR-15a-5p	1,218	1,903	-0.8	2.65×10^{-3}
miR-27a-3p	95,884	148,173	-0.9	2.17×10^{-6}
miR-505-3p	948	1,559	-0.9	4.57×10^{-5}
miR-26b-5p	88,613	195,026	-0.9	5.07×10^{-5}
miR-200c-3p	690	1,418	-0.9	1.38×10^{-3}
miR-16-5p	24,399	50,438	-1.0	5.56×10^{-6}
miR-19a-3p	2,369	4,147	-1.0	2.65×10^{-3}
miR-3613-5p	1,528	2,782	-1.0	1.09×10^{-3}
let-7c-5p	5,042	9,627	-1.0	1.12×10^{-3}
miR-374a-5p	5,224	12,467	-1.0	1.45×10^{-4}
miR-29a-3p	62,666	143,823	-1.1	9.84×10^{-6}
miR-16-2-3p	3,284	4,649	-1.1	1.03×10^{-3}
miR-126-3p	382,667	670,217	-1.1	9.4×10^{-4}
miR-142-5p	121,108	297,734	-1.2	8.24×10^{-3}
miR-214-5p	1,392	1,960	-1.2	3.09×10^{-3}
miR-194-5p	2,016	5,010	-1.3	9.64×10^{-10}
miR-140-5p	6,807	16,406	-1.4	2.61×10^{-7}
miR-17-3p	133	304	-1.4	1.09×10^{-3}
miR-30c-5p	26,531	70,312	-1.4	1.78×10^{-17}
miR-328-3p	1,757	4,171	-1.4	2.69×10^{-7}
miR-615-3p	692	1,024	-1.4	9.56×10^{-3}
miR-28-5p	2,279	6,041	-1.5	2.15×10^{-8}
miR-141-3p	124	454	-1.5	9.56×10^{-3}
miR-339-5p	1,579	4,150	-1.5	1.42×10^{-12}
miR-181c-5p	117	343	-1.5	9.11×10^{-3}
miR-125b-5p	18,480	43,337	-1.6	1.29×10^{-5}
miR-628-5p	50	178	-1.6	2.78×10^{-5}
miR-504-5p	373	913	-1.6	9.56×10^{-3}
miR-874-3p	611	1,974	-1.7	2.06×10^{-7}
miR-10b-3p	219	517	-1.7	2.91×10^{-4}
miR-142-3p	92,104	297,052	-1.7	1.17×10^{-8}
miR-342-5p	820	2,716	-1.7	1.26×10^{-3}
miR-1277-5p	31	107	-1.8	7.77×10^{-5}
miR-497-5p	674	2,116	-2.0	1.32×10^{-6}
miR-195-5p	2,637	8,364	-2.0	4.67×10^{-11}
miR-10398-3p	8	46	-2.1	8.24×10^{-3}
miR-125a-5p	22,706	75,239	-2.1	2.17×10^{-9}
miR-31-5p	254	1,229	-2.1	1.78×10^{-3}
miR-9983-3p	25	119	-2.3	6.71×10^{-3}
miR-99a-3p	7	34	-2.5	2.12×10^{-3}
miR-135a-5p	26	604	-2.5	6.82×10^{-3}

miR-129-5p	47	130	-2.7	1.09×10^{-3}
miR-4683-3p	1	23	-2.7	2.44×10^{-3}
miR-184-3p	10	98	-2.8	1.32×10^{-3}

Supplementary Table 3. Results of miR-214-5p *in situ* hybridization (ISH) assessment.

Case	ALK+ ALCL type	miR-214-5p ISH score	miR-214-5p expression*
#1	Common	score 3	High
#2	Common	score 2	High
#3	Common	score 3	High
#4	Common	score 1	Low
#5	Common	score 3	High
#6	Common	score 2	High
#7	Small cell	score 0	Low
#8	Small cell	score 0	Low
#9	Lympho-histiocytic	score 0	Low
#10	Lympho-histiocytic	score 1	Low
#11	Lympho-histiocytic	score 0	Low
#12	Lympho-histiocytic	score 0	Low

*differences in the distribution of expression groups (i.e. High/Low) between ALCL with common and variant histology (*i.e.* Small cell and Lympho-histiocytic) were statistically significant ($p = 0.02$).

References

1. Gaffo E, Bortolomeazzi M, Bisognin A, Di Battista P, Lovisa F, Mussolin L, Bortoluzzi S. MiR&moRe2: A Bioinformatics Tool to Characterize microRNAs and microRNA-Offset RNAs from Small RNA-Seq Data. *Int J Mol Sci* (2020) **21**: doi:10.3390/ijms21051754

2. Bortoluzzi S, Bisognin A, Biasiolo M, Guglielmelli P, Biamonte F, Norfo R, Manfredini R, Vannucchi AM, AGIMM (Associazione Italiana per la Ricerca sul Cancro–Gruppo Italiano Malattie Mieloproliferative) Investigators. Characterization and discovery of novel miRNAs and moRNAs in JAK2V617F-mutated SET2 cells. *Blood* (2012) **119**:e120-130. doi:10.1182/blood-2011-07-368001

3. Martin M. Cutadapt removes adapter sequences from high-throughput sequencing reads. *EMBnet.journal* (2011) **17**:10–12. doi:10.14806/ej.17.1.200

4. Langmead B, Trapnell C, Pop M, Salzberg SL. Ultrafast and memory-efficient alignment of short DNA sequences to the human genome. *Genome Biology* (2009) **10**:R25. doi:10.1186/gb-2009-10-3-r25

5. Love MI, Huber W, Anders S. Moderated estimation of fold change and dispersion for RNA-seq data with DESeq2. *Genome Biology* (2014) **15**:550. doi:10.1186/s13059-014-0550-8

6. Leek JT, Johnson WE, Parker HS, Jaffe AE, Storey JD. The sva package for removing batch effects and other unwanted variation in high-throughput experiments. *Bioinformatics* (2012) **28**:882–883. doi:10.1093/bioinformatics/bts034

7. van Balkom BW, de Jong OG, Smits M, Brummelman J, den Ouden K, de Bree PM, van Eijndhoven MA, Pegtel DM, Stoorvogel W, Würdinger T, Verhaar MC. Endothelial cells require miR-214 to secrete exosomes that suppress senescence and induce angiogenesis in human and mouse endothelial cells. Blood. 2013 May 9;121(19):3997-4006, S1-15.
